# New Value of *Acorus tatarinowii*/*gramineus* Leaves as a Dietary Source for Dementia Prevention

**DOI:** 10.3390/nu16111589

**Published:** 2024-05-23

**Authors:** Tomohiro Umeda, Ayumi Sakai, Keiko Shigemori, Kunio Nakata, Ryota Nakajima, Kei Yamana, Takami Tomiyama

**Affiliations:** 1 Department of Translational Neuroscience, Osaka Metropolitan University Graduate School of Medicine, 1-4-3 Asahimachi, Abeno-ku, Osaka 545-8585, Japan; ume@omu.ac.jp (T.U.);; 2 Cerebro Pharma Inc., 4-5-6-3F Minamikyuhojimachi, Chuo-ku, Osaka 541-0058, Japan; 3 NOMON Co., Ltd., New Business Development Unit, Teijin Ltd., Kasumigaseki Common Gate West Tower 3-2-1 Kasumigaseki, Chiyoda-ku, Tokyo 100-8585, Japan; nakata@nomon.jp (K.N.); nakajima@nomon.jp (R.N.); yamana@nomon.jp (K.Y.)

**Keywords:** *Acorus tatarinowii* Schott, *Acorus gramineus* Solander, Alzheimer’s disease, dementia with Lewy bodies, frontotemporal dementia, prevention

## Abstract

The rhizomes of *Acorus tatarinowii* Schott and *Acorus gramineus* Solander are widely used for treating amnesia in traditional Chinese medicine. In contrast, their leaves are usually discarded without their medicinal properties being known. Here, we found that the hot water extract of leaves improved cognition and tau pathology in model mice of frontotemporal dementia, similar to or even better than that of rhizomes. To explore the optimal method of processing, we made three preparations from dried leaves: hot water extract, extraction residue, and non-extracted simple crush powder. Among them, the simple crush powder had the strongest effect on tauopathy in mice. The crush powder also ameliorated Aβ and α-synuclein pathologies and restored cognition in mouse models of Alzheimer’s disease and dementia with Lewy bodies. These findings suggest the potential of *Acorus tatarinowii*/*gramineus* leaves as a dietary source for dementia prevention and reveal that simple crushing is a better way to maximize their efficacy.

## 1. Introduction

Dementia is an age-related disorder whose prevalence is increasing rapidly worldwide. The main cause of dementia is neurodegenerative diseases, which include Alzheimer’s disease (AD), frontotemporal dementia (FTD), and dementia with Lewy bodies (DLB). They are characterized by the cerebral accumulation of amyloidogenic proteins: Aβ and tau in AD, tau in FTD, and α-synuclein in DLB [1,2]. When aggregated to form oligomers, these proteins have been shown to exhibit synaptic toxicity [3,4] and propagate between cells [5,6]. Clinical studies have revealed that the accumulation of these proteins begins decades before the onset of dementia and that many neurons die by the time clinical symptoms emerge [7,8]. These findings demonstrate the importance of early diagnosis and prevention. However, presymptomatic diagnosis of dementia has not yet been established, and no inexpensive and safe preventive medicine that can be taken by all asymptomatic and undiagnosed people for a long period at their own discretion has yet been developed. Furthermore, prophylactic drugs must be broadly effective against etiologic proteins and capable of repairing neurons damaged by toxic oligomers. These requirements are difficult to meet with single-ingredient pharmaceuticals but may be feasible with proper diets composed of multiple ingredients.

Exploring a source for such preventative diets, we noticed two medicinal plants, *Acorus tatarinowii* Schott and *Acorus gramineus* Solander. They belong to the genus *Acorus* and have similar morphological characteristics in their aerial parts (i.e., leaves) and rhizomes [9]. Their rhizomes, *Acorus tatarinowii* rhizome (ATR) and *Acorus gramineus* rhizome (AGR), are widely used for amnesia in traditional Chinese medicine [10,11,12]; ATR is used in China, and AGR is used in Japan [9]. In contrast, their leaves, referred to as *Acorus tatarinowii* leaf (ATL) and *Acorus gramineus* leaf (AGL), are seldom used in traditional Chinese medicine and are usually discarded without their medicinal properties being known. In Japan, the rhizomes are treated as pharmaceuticals, whereas the leaves are regarded as non-pharmaceuticals (i.e., treatable as foodstuffs). The rhizomes contain various ingredients [10,11,12], and α-asarone and β-asarone are the main active components that have been shown to improve cognition in mice and rats [13,14,15,16,17,18,19]. We speculated that the leaves may contain some active ingredients similar to the rhizomes; therefore, we compared their effects on mouse cognition. Here, we showed that the hot water extract of AGL improved cognition and attenuated neuropathology in Tau784 mice, a model of FTD [20,21], similar to or even better than AGR. In traditional Chinese medicine, medicinal plants are often subjected to decoction, and the extraction residues are usually discarded. However, the residues may also contain some functional ingredients, and the hot water extraction process may break down heat-sensitive components and cause the loss of volatile substances by evaporation. To test this possibility, we made three preparations from dried ATL: hot water extract, extraction residue, and non-extracted simple crush powder, and we measured their effects on Tau784 mice. The extraction residue showed the same efficacy as the hot water extract, and the simple crush powder had the strongest effect. The crush powder also ameliorated Aβ and α-synuclein pathologies in APP23 [22,23,24] and Huα-Syn (A53T) mice [25,26], which are models of AD and DLB, respectively. Component analysis revealed that α-asarone and β-asarone were significantly lost during hot water extraction. Our findings shed light on the previously unknown value of *Acorus tatarinowii/gramineus* leaves as a dietary source for dementia prevention and reveal that simple crushing is a better way to maximize the medicinal effects of dried herbs.

## 2. Materials and Methods

### 2.1. Preparation of Hot Water Extracts of AGR and AGL

Hot water extracts of AGR and AGL were prepared at Maruzen Pharmaceuticals Co., Ltd. (Onomichi, Japan). Briefly, the dried rhizomes (500 g) and leaves (400 g) of *Acorus gramineus* Solander (origin: Hubei Province, China) were cut into small pieces and added to 8 and 30 volumes (*v*/*w*) of water, respectively. The suspensions were heated at 90 °C for 1 h and passed through 32-mesh and then 200-mesh filters. The filtrates were recovered, and the residues were heat-extracted again. The two extraction filtrates were combined and condensed into a viscous solution by lyophilization. The yields of extracts were 98 g for AGR and 130 g for AGL, which contained 52.2% and 50.0% solid matter by weight, respectively.

### 2.2. Preparation of Hot Water Extract, Extraction Residue, and Simple Crush Powder of ATL

Hot water extract of ATL was prepared at Auropure LifeScience Co., Ltd. (Zhuzhou, China). Dried ATL (origin: Hubei Province, China) was cut into small pieces and added to 8 volumes (*v*/*w*) of water. The suspensions were boiled for 1 h and passed through a filter. As an excipient, dextrin was added to the filtrates at a ratio of 80 parts solid matter to 20 parts dextrin. The mixture was spray-dried and put through a 60-mesh sieve. The passed-through powder was collected as a hot water extract. Non-extracted simple crush powder and extraction residue of ATL were prepared in our laboratory. Dried ATL (5.3 kg), which was obtained from Auropure LifeScience, was ground using a cutter mill and passed through a 2 mm screen. The obtained crude powder (5.1 kg) was sterilized at 170 °C for 3 s, crushed with a ball mill, and put through a vibrating sieve (500 μm of mesh). The passed-through powder was collected as a simple crush powder (4.0 kg). To prepare the extraction residue, the simple crush powder was added to 14 volumes of water, heated at 90 °C for 3 h, and put through a 5 μm filter. The residue on the filter was dried at 40 °C under reduced pressure and collected as an extraction residue.

### 2.3. Component Analysis of Acorus Preparations

Acorus rhizomes are known to contain α-asarone, β-asarone [13,14,15,16,17,18,19], and eugenol [27] as major active ingredients. The analysis of these components in our Acorus preparations was performed at Japan Food Research Laboratories (Tokyo, Japan). Dried ATR and ATL (origin: Hunan Province, China) were obtained from Auropure LifeScience and cut into small pieces in our laboratory. These materials and the above ATL hot water extract were sent to Japan Food Research Laboratories, where simple crush powders of ATR and ATL were prepared. One gram of each test sample was suspended in 20 mL of water, 10 mL of diethyl ether, and 8 g of sodium chloride and shaken for 10 min. After centrifugation, the supernatant was collected and analyzed by gas chromatography-mass spectrometry using an Agilent 5977B MSD (mass selective detector) with a 7890N GC (gas chromatograph) and DB-WAX UI GC column (all from Agilent Technologies, Wilmington, DE, USA). The estimated m/z value is 208 for α-/β-asarone and 164 for eugenol.

### 2.4. Mice

Three different mouse models of neurodegenerative dementia were used: Tau784 as a model of FTD, APP23 as a model of AD, and Huα-Syn (A53T) G2-3 line as a model of DLB. Tau784 mice exhibit tau hyperphosphorylation, tau oligomer accumulation, synapse loss, and memory impairment at 6 months [20,21]. APP23 mice show Aβ oligomer accumulation, synapse loss, memory impairment, and amyloid deposition by 15 months [22,23,24]. Huα-Syn (A53T) mice exhibit α-synuclein phosphorylation, α-synuclein oligomer accumulation, synapse loss, and memory impairment at 6 months [25,26]. All transgenic mice were maintained and used as heterozygotes. The mice were individually housed, and after reaching an age at which neuropathology and cognitive deficits could be reliably observed, they were divided into several groups with equal average body weights and equal numbers of males and females.

### 2.5. Treatment of Mice

The herbal preparations were orally administered to mice 5 days a week from Monday to Friday for 1 month. To compare the effects of AGR and AGL, liquid extracts containing about 50 weight % solid matter were diluted to 2.5 mg/mL in water. A total of 400 μL of the solutions (i.e., 0.5 mg solid matter) was administered to Tau784 mice. The same volume of water was administered to age-matched Tg and non-Tg littermates as controls. To compare the effects of the three preparations, ATL hot water extract, extraction residue, and non-extracted simple crush powder were suspended in water to 0.33 mg/mL by sonication. A total of 300 μL of each suspension (i.e., 0.1 mg of each preparation) was administered to Tau784 mice. To study the effects of ATL simple crush powder on APP23 and Huα-Syn (A53T) mice, 300 μL of solution containing 0.1 mg of powder was administered.

### 2.6. Behavioral Test

The cognitive function of mice was examined using the Morris water maze, as described previously [24]. AGR, AGL, and ATL treatments were continued during the behavioral test.

### 2.7. Histological Analysis of Neuropathology, Brain-Derived Neurotrophic Factor (BDNF) Expression, and Neurogenesis

After the water maze tests, half of the mice in each group were assigned for histological examination of neuropathology, BDNF expression, and neurogenesis, and the other half for future biochemical analysis. Brain sections were prepared as described previously [24] and stained with the following antibodies: AT8 (Thermo Scientific, Waltham, MA, USA) for phosphorylated tau, TOMA-1 (Merck Millipore, Darmstadt, Germany) for tau oligomers, 11A1 (IBL, Fujioka, Japan) for Aβ oligomers, β001 [24] for amyloid deposits, EP1536Y (Abcam, Cambridge, UK) for S129-phosphorylated α-synuclein, Syn33 (Sigma-Aldrich, St Louis, MO, USA), for α-synuclein oligomers, SVP-38 (Sigma-Aldrich) for synaptophysin, and GTX132621 (GeneTex, Irvine, CA, USA) for BDNF. The staining intensity or positive area in a constant brain region was quantified using NIH ImageJ software (ImageJ bundled with 64-bit Java 8). Neurogenesis was assessed as described previously [28]. In brief, 5-bromo-2′-deoxyuridine (BrdU; Sigma-Aldrich) was injected into the mice for the last 5 days of ATL treatment. Brain sections were stained with anti-BrdU (IBL) and anti-doublecortin (DCX) antibodies (Abcam), and positive cells for both BrdU and DCX were regarded as newly generated neurons and counted in a constant brain region.

### 2.8. Statistical Analysis

All experiments and data analyses were performed under unblinded, open-label conditions. In a comparison of more than two groups, we used analysis of variance (ANOVA) followed by Fisher’s PLSD as a post hoc test. For the behavioral tests, two-factor repeated measures ANOVA was used.

## 3. Results

### 3.1. Comparison of Hot Water Extracts of AGR and AGL in Tau784 Mice

**Figure 1:**
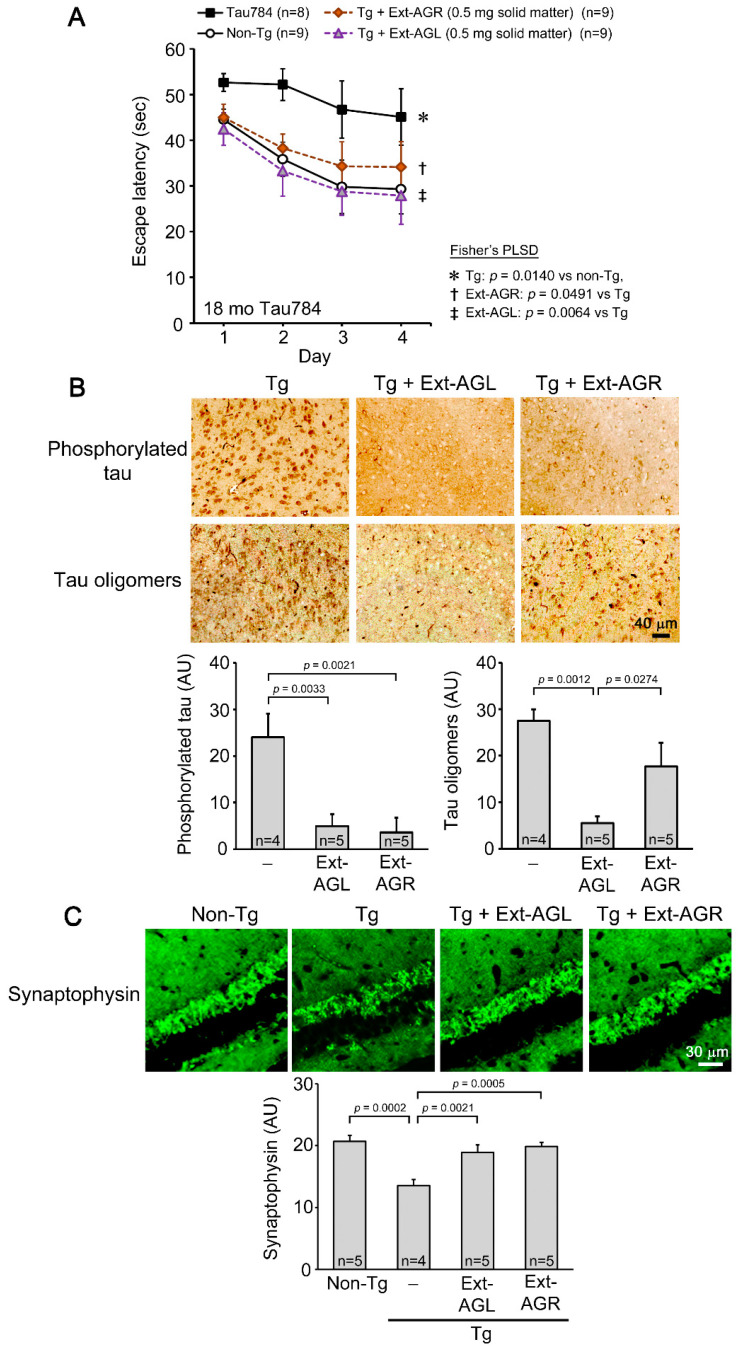
Comparison of hot water extracts of AGR and AGL in Tau784 mice. Hot water extracts (Ext) of AGR and AGL were administered to 17-month-old Tau784 mice at 0.5 mg solid matter/shot for 1 month. (**A**) Mouse memory was significantly improved by both extracts, but Ext-AGL had a stronger effect. (**B**) The levels of phosphorylated tau and tau oligomers in the entorhinal cortex were significantly reduced by both extracts, but again, the effect of Ext-AGL was significantly stronger. (**C**) Synaptophysin levels in the hippocampal CA3 region were significantly restored by both extracts to levels similar to those in non-Tg littermates.

To study the potential of Acorus leaves, we initially compared the effects of AGR and AGL in a mouse model of FTD. Hot water extracts of AGR and AGL containing 0.5 mg of solid matter each were administered to 17-month-old Tau784 mice (mean body weight, 36.1 g) for 1 month. In the water maze tests, both extracts significantly improved mouse memory, but AGL showed an unexpectedly stronger effect (Figure 1A). Tau pathology was examined in the entorhinal cortex by immunohistochemistry. Both extracts significantly reduced the level of phosphorylated tau and tau oligomers, but again, the effect of AGL was significantly stronger (Figure 1B). Synaptophysin levels in the hippocampal CA3 region were restored by both extracts to levels similar to those in non-Tg littermates (Figure 1C). To our knowledge, these results are the first evidence showing that AGL can improve cognition comparably to or even better than AGR. Thus, we focused on the leaves hereafter.

### 3.2. Comparison of Hot Water Extract, Extraction Residue, and Simple Crush Powder of ATL in Tau784 Mice

**Figure 2:**
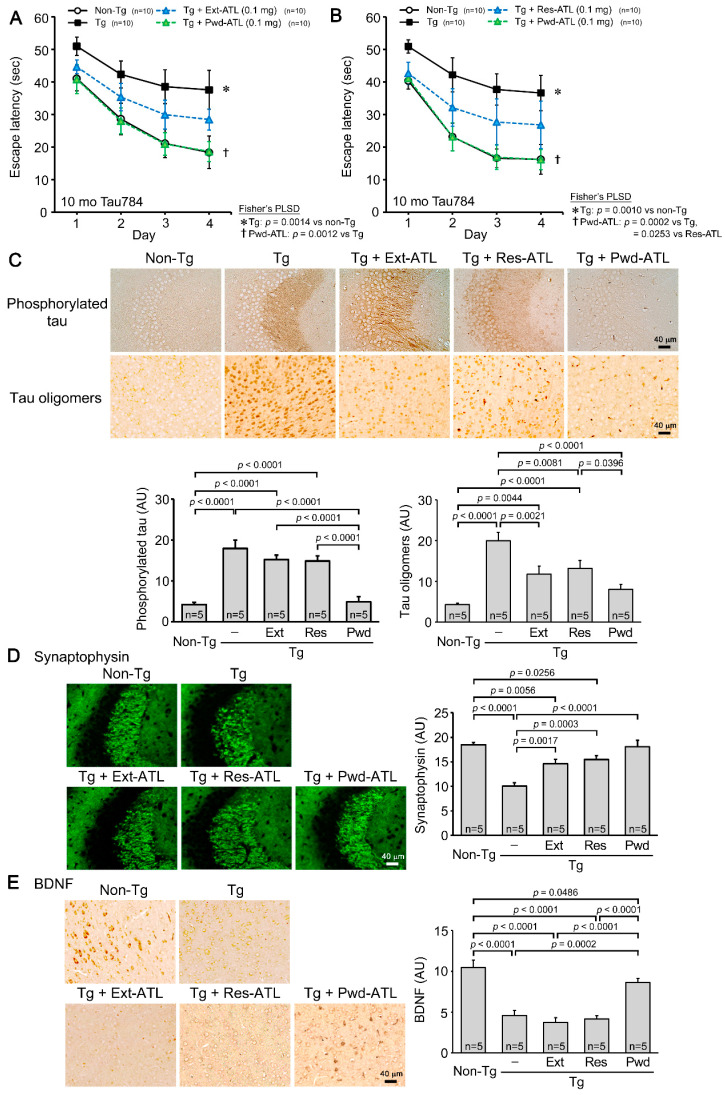
Comparison of hot water extract, extraction residue, and non-extracted simple crush powder of ATL in Tau784 mice. Hot water extract (Ext) and simple crush powder (Pwd) of ATL were administered to 9-month-old Tau784 mice at 0.1 mg/shot for 1 month. Subsequently, Pwd and extraction residue (Res) of ATL were administered to 8- to 9-month-old Tau784 mice at 0.1 mg/shot for 1 month. (**A**) Pwd-ATL rescued mouse memory to the same level as that of non-Tg littermates, while the effect of Ext-ATL was incomplete. (**B**) Again, Pwd-ATL rescued mouse memory to the same level as that of non-Tg littermates, while the effect of Res-ATL was incomplete, similar to that of Ext-ATL. (**C**) The levels of phosphorylated tau and tau oligomers were attenuated by all three preparations, with Pwd-ATL showing the strongest effects. (**D**) Synaptophysin levels were significantly restored by all three preparations, but only Pwd-ATL had almost complete effects. (**E**) BDNF expression in the cerebral cortex significantly decreased in Tg mice. Pwd-ATL significantly restored BDNF expression, albeit to a level slightly lower than that in non-Tg littermates, while Ext-ATL and Res-ATL had no effect.

Next, to explore the optimal method of processing, we compared the effects of hot water extract, extraction residue, and simple crush powder of ATL. The hot water extract and simple crush powder were administered to 9-month-old Tau784 mice (mean body weight, 28.5 g) at 0.1 mg/shot for 1 month. The hot water extract improved mouse memory, but the effect was incomplete at this dose (Figure 2A). In contrast, the simple crush powder completely rescued mouse memory to the same level as that of non-Tg littermates. Subsequently, the simple crush powder and extraction residue were administered to 8- to 9-month-old Tau784 mice (mean body weight, 30.1 g) at 0.1 mg/shot for 1 month. Once more, the simple crush powder achieved complete recovery (Figure 2B). The extraction residue, on the other hand, had a moderate effect, similar to that of the hot water extract. The level of phosphorylated tau was significantly attenuated by the simple crush powder but only slightly attenuated by the hot water extract or extraction residue (Figure 2C). The level of tau oligomers was significantly reduced by all three preparations, but the simple crush powder had the strongest effect (Figure 2C). Synaptophysin levels in the hippocampal CA2/3 region were significantly restored by all three preparations, but only the simple crush powder restored them to levels similar to those in non-Tg littermates (Figure 2D). These results support our speculation that some functional ingredients remain in the extraction residue and that the decoction process results in the loss of some functional components due to thermal destruction and evaporation. Next, we evaluated whether ATL can repair neurons damaged by toxic oligomers. We measured the expression levels of BDNF in the cerebral cortex by immunohistochemistry, because BDNF plays an important role in repairing neurons [29]. Compared to their non-Tg littermates, Tau784 mice showed a significant decrease in BDNF expression (Figure 2E). The simple crush powder significantly restored BDNF expression to a level slightly lower than that in non-Tg littermates. In contrast, the hot water extract and extraction residue had no effect on BDNF.

### 3.3. Component Analysis of Acorus Preparations

α-Asarone, β-asarone [13,14,15,16,17,18,19], and eugenol [27] are well-known components of Acorus rhizomes that improve cognitive function. We speculated that Acorus leaves also contain these components at levels comparable to those of the rhizomes; therefore, AGL exhibited nootropic activity similar to that of AGR. Furthermore, given that these substances are volatile oils, hot water extraction may result in the loss of these components through evaporation, which is why the decoction sample showed weaker activity than the simple crush powder. To test these hypotheses, we examined the content of these ingredients in simple crush powders of ATR and ATL and in a hot water extract of ATL. As shown in Table 1, the concentrations of these components were much lower than our expectations in the leaves and markedly decreased to the detection limit after hot water extraction. These results suggest that the leaves contain other active substances in addition to α-asarone, β-asarone, and eugenol and that to maximize the medicinal effects of dried herbs, simple crushing is better than hot water extraction.

### 3.4. Effects of ATL Simple Crush Powder on APP23 Mice

**Figure 3:**
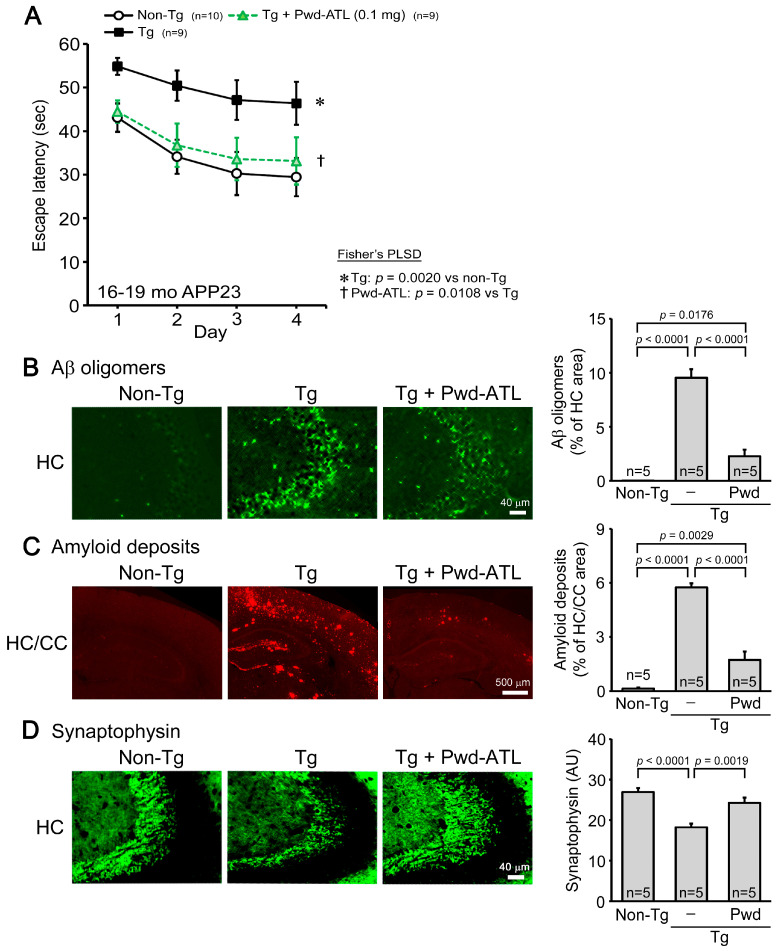
Effects of ATL simple crush powder on APP23 mice. ATL simple crush powder (Pwd-ATL) was administered to 15- to 18-month-old APP23 mice at 0.1 mg/day for 1 month. (**A**) Mouse memory was significantly improved to a level similar to that of non-Tg littermates. The levels of Aβ oligomers (**B**) and amyloid deposits (**C**) in the hippocampus (HC) and cerebral cortex (CTX) were significantly reduced. (**D**) Synaptophysin levels in the hippocampal CA2/3 region were significantly restored to a level similar to that in non-Tg littermates.

Next, we tested the effects of ATL simple crush powder on a mouse model of AD. Simple crush powder was administered to 15- to 18-month-old APP23 mice (mean body weight, 28.8 g) at 0.1 mg/shot for 1 month. Mouse memory was significantly improved by the treatment to a level similar to that of non-Tg littermates (Figure 3A). Aβ pathologies were examined in the cerebral cortex and hippocampus by immunohistochemistry. The levels of Aβ oligomers (Figure 3B) and amyloid deposits (Figure 3C) were significantly reduced in both regions. Synaptophysin levels in the hippocampal CA2/3 region were significantly restored to a level similar to that of non-Tg littermates (Figure 3D).

### 3.5. Effects of ATL Simple Crush Powder on Huα-Syn (A53T) Mice

**Figure 4:**
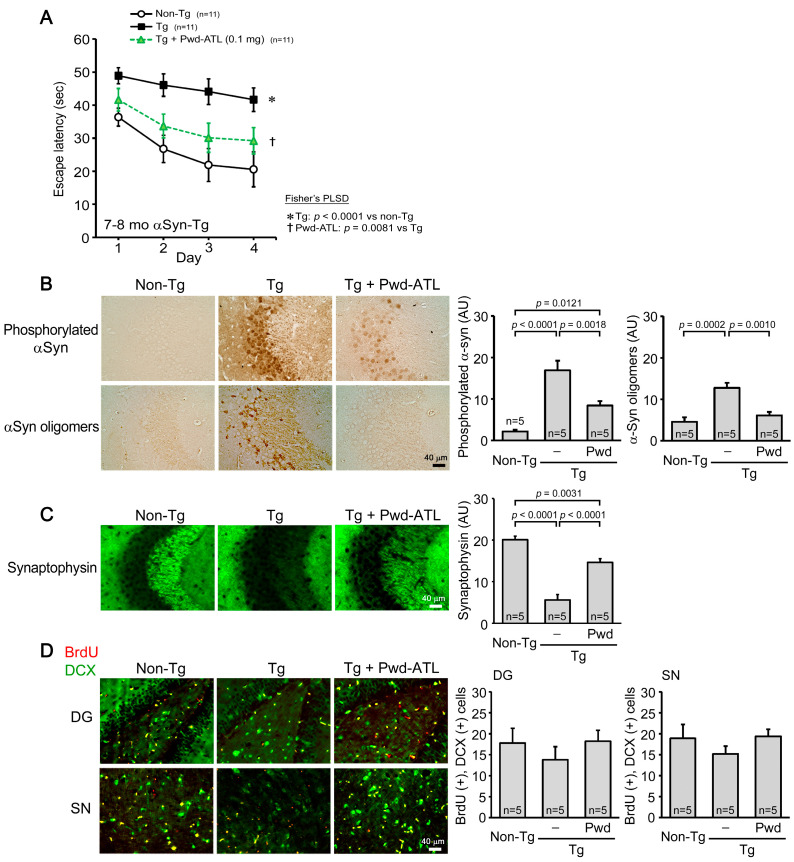
Effects of ATL simple crush powder on Huα-Syn (A53T) mice. ATL simple crush powder (Pwd-ATL) was administered to 6- to 7-month-old Huα-Syn (A53T) mice at 0.1 mg/day for 1 month. (**A**) Mouse memory was significantly improved by the treatment, but the level did not reach that of non-Tg littermates. (**B**) The levels of phosphorylated α-synuclein and α-synuclein oligomers in the hippocampus (HC) and entorhinal cortex (EC) were significantly reduced. (**C**) Synaptophysin levels in the hippocampal CA2/3 region were significantly restored to a level similar to that in non-Tg littermates. (**D**) Neurogenesis in the dentate gyrus (DG) and substantia nigra (SN) of Tg mice was recovered by Pwd-ATL to levels similar to those in non-Tg littermates. Positive cells for both BrdU (red) and DCX (green) were regarded as newly generated neurons.

Finally, we examined the effects of ATL simple crush powder on a mouse model of DLB. Simple crush powder was administered to 6- to 7-month-old Huα-Syn (A53T) mice (mean body weight, 28.0 g) at 0.1 mg/shot for 1 month. Mouse memory was significantly improved by the treatment, but the level did not reach that of non-Tg littermates (Figure 4A). α-Synuclein pathologies were assessed in the hippocampus by immunohistochemistry. The levels of phosphorylated α-synuclein and α-synuclein oligomers were significantly reduced by the treatment (Figure 4B). Synaptophysin levels in the hippocampal CA2/3 region were significantly restored to a level similar to that of non-Tg littermates (Figure 4C). In addition to BDNF, neurogenesis participates in repairing neurons [30]. Thus, we investigated the effects of ATL powder on neurogenesis in the dentate gyrus and substantia nigra. Compared to non-Tg littermates, Tg mice showed a decrease in neurogenesis in both brain regions, and their levels were recovered by ATL powder to levels similar to those in non-Tg littermates.

## 4. Discussion

Dementia prophylactic agents should be (1) highly safe, (2) inexpensive, (3) non-invasively taken by people at their own discretion, (4) broadly effective against etiologic proteins, and (5) capable of repairing neurons damaged by toxic oligomers. To identify the ingredients that meet these requirements, we explored the medicinal herbs used in traditional Chinese medicine. Several herbs have been reported to have beneficial effects on amnesia, including *Rehmanniae radix* [31], *Polygalae radix* [32], *Zizyphi spinosi* semen [33], and ATR/AGR [10,11,12]. When considering these herbs as foodstuffs, we need to be especially careful about their safety because they will be consumed for a long period. Of the herbs listed above, only *Zizyphi spinosi* semen is treated as a non-pharmaceutical (i.e., treatable as food), while the others are considered pharmaceuticals in Japan. However, *Zizyphi spinosi* semen is mainly harvested in China, and its procurement may be expensive. On the other hand, while ATRs/AGRs are regarded as pharmaceuticals, their leaves are treated as non-pharmaceuticals in Japan, indicating their safety. The leaves are usually discarded without being used in traditional Chinese medicine, and their procurement costs are very low. Since both the rhizomes and leaves come from the same plant, we thought that the leaves might have the same medicinal properties as the rhizomes. In the present study, we found that AGL extract is as effective as or even more effective than AGR extract at improving mouse cognition. In addition, ATL was broadly effective against Aβ, tau, and α-synuclein and was capable of repairing neurons through BDNF expression and neurogenesis. These findings not only reveal a previously unknown value of ATL/AGL but also provide a way to produce dementia preventive diets at low procurement costs. Furthermore, our results demonstrate that simple crushing rather than hot water extraction can maximize the medicinal effects of ATL, which may also lower manufacturing costs.

While our study suggests several advantages of multi-ingredient diets, the identification of the active components in ATL/AGL would facilitate the development of pharmaceuticals for treating neurodegenerative diseases. ATR and AGR are known to contain various ingredients [10,11,12], among which α-asarone and β-asarone have been well studied [13] and are reported to improve cognition in animal models of neurodegenerative dementia. For example, oral administration of α-asarone improves cognition in Aβ25-35-injected rats by reducing nitric oxide levels [14]. Intraperitoneal injection of α-asarone also improves cognition in APP/PS1 mice by reducing the levels of Aβ42, phosphorylated tau, and neuroinflammation and by promoting neuronal survival in the hippocampus [15]. Oral administration of β-asarone improves cognition in APP/PS1 mice by inhibiting neuronal apoptosis via activation of the CaMKII/CREB/Bcl-2 pathway [16], reducing Aβ42 levels via activation of Beclin-1-dependent autophagy [17], and increasing the levels of synaptophysin and glutamatergic receptor 1 [18]. Oral β-asarone also improves cognition in Aβ42-injected rats by regulating PINK1-Parkin-mediated mitophagy [19]. In addition to these components, eugenol has also been reported to have beneficial effects on cognition. The oral administration of eugenol mitigates cognitive impairments in 5 × FAD mice by reducing Aβ deposition and neuronal loss and increasing microglial phagocytosis [27]. Based on these studies and our results in Figure 1, we speculated that these active components are present at similar levels in both the rhizomes and leaves. However, the component analysis revealed that the content of α-asarone, β-asarones, and eugenol in the ATL powder was much lower than that in the ATR powder (Table 1), implying that other active substances contribute to the activities of ATL. While these volatile components were drastically reduced by hot water extraction (Ext-ATL in Table 1), the nootropic activity of the hot water extract was not reduced as dramatically (Figure 2A). Furthermore, the extraction residue had nootropic activity comparable to that of the hot water extract (Figure 2B). These results collectively suggest that the active components in ATL include non-volatile and insoluble substances. These may be dietary fibers, which are presumably contained at higher levels in the extraction residue and simple crush powder than the hot water extract. It has been suggested that soluble and insoluble dietary fibers derived from plants affect brain function through gut microbes by regulating the intestinal environment [34,35].

It has been suggested that certain diets are effective in preventing dementia. For example, the Mediterranean diet, which is comprised primarily of vegetables, fruits, nuts, legumes, unrefined grains, fish, and olive oil, has been shown to lower the risk of cognitive decline and dementia thanks to its neuroprotective ingredients such as polyunsaturated fatty acids, polyphenols, and antioxidants [36,37,38]. However, it is not easy for ordinary people to adhere to the Mediterranean diet in their daily meals. If it were in the form of a dietary supplement that is more specific to dementia, it would be easier to continue and introduce into daily life. As a nootropic supplement prepared from a single plant, Ginkgo biloba extract has been well studied. Ginkgo biloba extract contains many terpenoids such as ginkgolides, which promote blood flow, and flavonoids such as quercetin, which has antioxidant properties, and exhibits neuroprotective and anti-inflammatory effects [39]. Intraperitoneal injection of EGb761 (Ginkgo biloba extract) at 30 mg/kg/day for 4 months improves cognition in 5 × FAD mice by reducing Aβ deposits and promoting neurogenesis [40]. Oral administration of EGb761 at 50 mg/kg/day for 6 months also improves cognition in APP/PS1 mice by inhibiting amyloid plaque deposition and downregulating pro-inflammatory cytokines [41]. This oral dosage and duration are higher and longer than those of ATL, although the mouse model used is different. A meta-analysis of clinical studies suggests that EGb761 may improve cognition in early-stage AD patients after high doses (240 mg/day) and long-term (over 24 weeks) administration [42]. Previously, we showed that the leaves of the Hawaiian herb Mamaki have strong effects on improving neuropathology and cognitive function in mouse models of neurodegenerative dementia [28]. Mamaki leaves contain a lot of polyphenols, such as catechins, chlorogenic acid, and rutin, and exhibit strong antioxidant effects [43]. However, our in vivo experiments revealed that the nootropic effects of Mamaki cannot be explained by these polyphenols alone. Furthermore, simply crushed powder was more effective than hot water extract of the leaves. Components other than polyphenols, such as dietary fibers again, may play an important role in the effects of Mamaki.

In the present study, we used the Morris water maze test in evaluating the cognitive function of mice, since the test is considered the most reliable one to measure long-term memory in animals. Performing additional behavioral tests would make the results more credible, but even after multiple examinations, mouse models do not fully represent human pathology; therefore, clinical studies are required to evaluate the true efficacy of ATL/AGL in humans. ATL/AGL can be treated as a non-pharmaceutical agent in Japan, suggesting its safety. However, some studies have revealed the possible toxicity of α- and β-asarone in chronic administration to animals [44,45]. In addition, *Acorus gramineus* and *Acorus tatarinowii* contain more than 160 compounds of different structural types, including phenylpropanoids, terpenoids, lignans, flavonoids, alkaloids, amides, organic acids, and others [10,11,12]. These compounds are thought to work together to exert the medicinal effects of the plants, but some of them may be toxic at a high concentration. Long-term intake of these compounds may lead to the accumulation of such toxicity and ultimately cause adverse events. Therefore, the safety of ATL/AGL simple crush powder should be carefully confirmed before human trials. Despite these challenges, the utilization of ATL/AGL would provide a simple (i.e., just crushing), inexpensive, and environmentally friendly (i.e., using the previously discarded material) way to make dementia preventive diets.

## 5. Conclusions

In the present study, we found that the leaves of *Acorus tatarinowii* Schott and *Acorus gramineus* Solander, which are usually discarded in traditional Chinese medicine, showed unexpectedly strong neuroprotective effects in mouse models of neurodegenerative dementia, including AD, FTD, and DLB. Among the three preparations made from dried leaves, hot water extract, extraction residue, and non-extracted simple crush powder, the last one had the strongest effect on improving neuropathology and cognition in mice. Although the active components in the sample remain to be identified, our findings suggest the novel value of *Acorus tatarinowii/gramineus* leaves as a food material and provide a simple, inexpensive, and environmentally friendly way to produce multi-ingredient diets that meet all the requirements for dementia prophylactic agents.

## Figures and Tables

**Table 1:** The three major components in each Acorus preparation.

Components	Pwd-ATR	Pwd-ATL	Ext-ATL
α-Asarone	39 ppm	9.8 ppm	<0.1 ppm
β-Asarone	760 ppm	120 ppm	0.1 ppm
Eugenol	6.0 ppm	0.7 ppm	0.2 ppm

Pwd-ATR, simple crush powder of ATR (rhizome); Pwd-ATL, simple crush powder of ATL (leaf); Ext-ATL, hot water extract of ATL.

## Data Availability

Data is available in a publicly accessible repository: https://cerebro-p.app.box.com/s/7s5fqhllxa5qc1dnqik1lc29gs7ok88i.
